# Dry but Not Humid Thermal Processing of *Aloe vera* Gel Promotes Cytotoxicity on Human Intestinal Cells HT-29

**DOI:** 10.3390/foods11050745

**Published:** 2022-03-03

**Authors:** Zaira López, Michelle N. Salazar Zúñiga, Antoni Femenia, Gustavo J. Acevedo-Hernández, Jaime A. Godínez Flores, M. Eduardo Cano, Peter Knauth

**Affiliations:** 1 Cell Biology Laboratory, Departamento de Ciencias Médicas y de la Vida, Centro Universitario de la Ciénega (CUCI), Universidad de Guadalajara (UdG), Avenida Universidad 1115, Ocotlan 47810, Jalisco, Mexico; zlopez@gmx.net (Z.L.); sazmn22@gmail.com (M.N.S.Z.); jagf1812@hotmail.es (J.A.G.F.); 2 Department of Chemistry, University of the Balearic Islands, Ctra. Valldemossa km 7.5, 07122 Palma de Mallorca, Spain; antoni.femenia@uib.es; 3 Laboratorio de Biología Molecular Vegetal, Departamento de Ciencias Médicas y de la Vida, Centro Universitario de la Ciénega (CUCI), Universidad de Guadalajara (UdG), Avenida Universidad 1115, Ocotlan 47810, Jalisco, Mexico; gacevedo72@gmail.com; 4 Laboratorio de Biofísica, Departamento de Ciencias Básicas, Centro Universitario de la Ciénega (CUCI), Universidad de Guadalajara (UdG), Avenida Universidad 1115, Ocotlan 47810, Jalisco, Mexico; meduardo2001@hotmail.com

**Keywords:** *Aloe vera*, acemannan, cytotoxicity, acetylation

## Abstract

*Aloe vera* products, both in food and cosmetics, are becoming increasingly popular due to their claimed beneficial effects, which are mainly attributed to the active compound acemannan. Usually, these end products are based on powdered starting materials. High temperatures during the drying process to obtain the starting materials have several advantages, like shortening the drying time, eliminating toxic aloin and reducing bacterial contamination. Nevertheless, there are two major drawbacks: first, at temperatures of 80 °C or higher, structural changes in acemannan, especially its deacetylation (>46%), are triggered, which does not happen at lower temperatures (14% at 60 °C); secondly, a toxic principle is formed at higher temperatures, resulting in a higher cytotoxicity. Thus, two temperature-dependent but opposing effects cause with a median cytotoxic concentration of CC_50_ = 0.4× a peak of cytotoxicity at 80 °C; at 60 °C this cytotoxic substance is not formed and at 100 °C aloin is more readily eliminated, resulting in a CC_50_ = 1.1× and CC_50_ = 1.4×, respectively. The cytotoxic substance generated by dry heat at 80 °C is not a modified polysaccharide because its polysaccharide-enriched alcohol-insoluble fraction is with CC_50_ = 0.9× less cytotoxic. Moreover, this substance is polar enough to be washed away with ethanol. Additionally, when Aloe gel is heated at 80 °C under humid conditions (pasteurization), the cytotoxicity does not increase (CC_50_ = 1.6×). Finally, to produce powdered starting materials from Aloe gel, it is recommended to use temperatures of around 60 °C in order to preserve the acemannan structure (and thus biological activity) and the low cytotoxicity.

## 1. Introduction

The succulent leaves from *Aloe vera* have an outer waxy cuticle and below a single cell epidermis, a thin layer of chlorenchyma cells. The vascular bundles produce a bitter tasting exudate (Aloe latex). The inner part consists of a thick watery parenchyma (Aloe fillet), which can be extruded obtaining the Aloe gel [1]. The Aloe latex contains most of the secondary metabolites, like glycosylated anthrones (e.g., aloin) or nonglycosylated anthraquinones (e.g., aloe emodin), which exhibit several toxic effects [2,3]. Therefore, most Aloe containing food or food supplements use Aloe gel or Aloe fillet. The latter consists of about 90–98% water; the residual dry mass is composed of approximately 57.6% crude fibers, 16.5% soluble saccharides, 15.4% ashes (minerals), 7.3% proteins and 4.2% lipids [4]. Physiological effects, especially immunomodulation, of Aloe fillet or Aloe gel are attributed to their polysaccharide fraction and in particular to acemannan [5,6]. *Aloe vera* acemannan, the major (50%) storage constituent [4,7], is composed out of mannose (>60%), glucose (~20%) and galactose (<10%). It forms a linear polysaccharide backbone of β-(1,4) mannose with β-(1,4) glucose interceptions and α-(1,6) galactosyl side-chains. Moreover, acetyl groups are linked at the C-2, C-3 and C-6 of mannose residues with a ratio of approximately one acetyl group per mannose residue [4,8,9,10]. It is known that the biological principle of polysaccharides, as well as other constituents, depend on their molecular structure; now, increasing evidence suggests that due to the thermal process used in the dehydration of *Aloe vera* gel, the acemannan may undergo important structural modifications [11,12,13,14].

When *Aloe vera* gel is dehydrated for commercial purpose at temperatures above 60 °C, the average molecular weight of acemannan increases from 45 kDa (fresh Aloe) to 75–81 kDa, which is attributed to losses of galactosyl residues and, also, to deacetylation processes, which, in turn, could contribute to hydrogen bonding between mannose chains, resulting in mannose-rich chains of higher molecular weight [13,14]. Any industrial drying process (spray-, freeze-, refractance window- or radiant zone-drying) causes deacetylation at >60% of the mannose units, while pasteurization increases the acemannan yield by ~40% [12]. Also after applying hot air at 50 °C, 60 °C or 70 °C with far-infrared radiation and a high-voltage electric field, the content of acemannans decreases by ~40–45% [15].

The acemannan polymer may exhibit several biological activities in humans like antitumour, anticancer, antioxidant and immunomodulatory activities [16]. In order to clarify whether thermal processes promote irreversible modifications in acemannan that affect their biological activity, several studies have recently been conducted. For instance, Chokboribal et al. (2015) [17] revealed that a decrease in the degree of acetylation reduced biological activity of fibroblasts in-vitro, such as DNA, VEGF and type I collagen synthesis. In addition, Hussain et al. (2019) [18] found that supplementation with heat-treated Aloe-juice (10 min at 70 °C, 80 °C or 15 min at 121 °C) reduced phagocytic activity of macrophages compared to a non-heated Aloe juice-control. Moreover, Salah et al. (2017) [19] reported that antibacterial activity (against *Staphylococcus aureus*, *Enterobacter faecalis*, *Escherichia coli*, *Pseudomonas aeruginosa*) decreased when deacetylation of acemannan increased. Generally, Aloe gel is not considered to be toxic or cytotoxic [20], but for these studies usually fresh or lyophilized Aloe gel samples were analysed. On the other hand, the industrial drying process and the resulting polysaccharide modifications may introduce cytotoxic effects: Du Plessis & Hamman [21] reported for an industrial 200× concentrated *Aloe vera* gel sample, containing much fiber, a cytotoxic concentration for HeLa cells of CC_50_ = 0.27 g/L (~0.05×) (authors [21] stated they prepared a stock solution with 10 g/L, which corresponds to a 2× concentrated solution for a 200× concentrated powder. From this stock solution dilutions ranging from 0.01 g/L to 1000 g/L were prepared. As this is impossible, we suppose a typo (mg/L instead of g/L)). Also, we have reported previously a cytotoxic effect for an industrial *Aloe vera* Inner Leaf gel sample with a high fiber content (Inner Leaf Fiber, ILF), a cytotoxic concentration of ~1 g/L (0.2×) for HeLa cells [22].

Starting materials for commercially available Aloe-containing foodstuff are usually powders, which are, depending on their drying process, dried at different temperatures. The aim of this work was to study the cytotoxic effects of heat-induced structural modifications, especially the percentage of deacetylation, of *Aloe vera* gel polysaccharides on the intestinal human cell line HT-29.

## 2. Materials and Methods

### 2.1. Plant Material Extraction

An US enterprise provided *Aloe vera*-powder, which is used by food supplemental industries as starting material: 200× dehydrated inner leaf gel with a high fiber content (ILF) [5,22]. This commercially available starting material was compared to fresh *Aloe vera* gel, dried at different temperatures. As control was used commercially available 200× lyophilized Aloe inner leaf gel (Inner Leaf gel Lyophilized, ILL) from Aloe Jaumave (Jaumave, Tamaulipas, Mexico).

Fresh whole leaves from *Aloe vera* plants grown on the campus of the University in Ocotlán (Jalisco, Mexico) were cut, which were washed and trimmed, then peeled and extensively washed with distilled water to remove the exudate from their surfaces (fillet). Then, 100 g of fillet were heated under humid conditions (pasteurized) in an autoclave at 60, 80, or 100 °C during 30 min (Pasteurized Aloe Gel, PAG60, PAG80 and PAG100) before the still humid samples were ground using an electrical grinder (model BLM10600G, Black & Decker, Towson, MD, USA). On the other hand, 100 g of fillet were dried in an electrical oven at 60, 80, or 100 °C during 15–20 h (Dehydrated Aloe Gel, DAG60, DAG80 and DAG100) achieving a yield of 2.9%, 2.2%, and 1.9%, respectively. Afterwards, the dry samples were ground too, and passed through a sieve with 30 mesh.

### 2.2. Molecular Identification through DNA Barcoding

DNA barcoding was used for the authentication of plant material used in this work. This method has been widely applied for the rapid and accurate identification of plants using DNA sequences [23]. Approximately 5 g of young fresh leaf gel was ground with a mortar and pestle under liquid nitrogen for homogenization, and 200 mg of the powdered material was used for DNA isolation using the GenElute Plant Genomic DNA Miniprep Kit (Sigma-Aldrich, St. Louis, MO, USA), following the protocol suggested by the manufacturer. The same procedure for DNA isolation was followed using 200 mg of the commercial powder (ILF).

The quality of the extracted genomic DNA was verified by agarose gel electrophoresis and the quantification was achieved by UV spectrophotometry using a Q5000 Spectrophotometer (Quawell, Sunnyvale, CA, USA). Primers and PCR conditions were as those reported by the Consortium for Barcode of Life (CBOL) Plant Working Group [23]. For amplification of *matK* gene, primers 3F_KIM_f (5′-CGT ACA GTA CTT TTG TGT TTA CGA G-3′) and 1R_KIM_r (5′-ACC CAG TCC ATC TGG AAA TCT TGG TTC-3′), and for amplification of *rbcL* gene primers rbcL*a*_R (5′-GTA AAA TCA AGT CCA CCR CG-3′) and rbcL*a*_F (5′-ATG TCA CCA CAA ACA GAG ACT AAA GC-3′) were used. Amplification reactions were performed in a final volume of 50 µL containing 20 mm Tris-HCl (pH 8.4), 50 mm KCl, 2 mm MgCl_2_, 0.2 mm dNTPs mix, 1 µM each primer, 20 ng of genomic DNA and 2.5 units of Taq DNA polymerase (Jena Bioscience, Jena, Germany). Amplifications were performed in a Multigene Thermal Cycler (Labnet International, Edison, NJ, USA) using the program: 1 step at 94 °C for 5 min, 35 cycles consisting of 30 s at 94 °C, 1 min at 52 °C and 1 min at 72 °C, with a final extension step of 5 min at 72 °C. The presence of single amplification products for each gene was verified by electrophoresis in 0.8% agarose gels and stained with EvaGreen (Jena Bioscience, Jena, Germany). PCR products were purified using PEG precipitation to remove residual primers and nucleotides [24]. The DNA concentration of the samples was estimated by spectrophotometry and PCR products were sent for sequencing to the Genomic Services Laboratory at Langebio-Cinvestav (Irapuato, Mexico). DNA sequences were used for plant identification by the Identification Engine at the Barcode of Life Data System (http://www.boldsystems.org/ (accessed on 1 June 2021)) and were submitted to the public sequence repository GenBank [25].

### 2.3. Alcohol Insoluble Residues

Alcohol insoluble residues (AIRs) were prepared from commercial (ILF) and from dehydrated (DAG60, DAG80 and DAG100) samples: the samples were suspended in boiling ethanol (85% *v*/*v*), homogeneized with an Ultra Turrax homogenizer T25 Digital (IKA, Staufen, Germany) (13,000 rpm for 1 min) and boiled for further 5 min as previously described in Waldron and Selvendran (1990) [26]. After cooling down, the mixture was rehomogenized, filtered through glass microfibre (Whatman, GF/C, 1.2 µm pore size) and re-extracted twice with boiling 85% (*v*/*v*) ethanol. Then the white residues were washed with absolute ethanol followed by acetone and air-dried overnight. Finally, the AIRs were milled using a laboratory type grain mill (M2 Universal Mill, IKA) and passed through a 0.5 mm aperture sieve. The AIRs samples were AIR of Inner Leaf Fiber (AILF) and AIR of dehydrated Aloe Gel (AAG60, AAG80 and AAG100). The control ILL was completely soluble; thus, it was not possible to prepare AIRs from this sample.

### 2.4. Analysis of Carbohydrate Composition

Dehydrated samples and AIR preparations (100 mg) were suspended in 200 mL distilled water and stirred for 2 h at room temperature. The suspensions were then centrifuged at 13,000× *g* for 15 min at 20 °C. The supernatants, containing the water-soluble polymer acemannan, and the precipitates, containing the cell wall polysaccharides (pectins, hemicellulose and cellulose), were collected and lyophilized. All extracts were stored in a desiccator under anhydrous conditions until later analysis [27].

Carbohydrate composition was analysed after acid hydrolysis following a modified version of the method reported by Rodríguez-González et al. (2011) [14]. *Aloe vera* samples (5 mg) were suspended in 12 M H_2_SO_4_ for 3 h, followed by dilution to 1 M and hydrolysed at 100 °C for 2.5 h (Saeman et al. 1954) [28]. A second sample, only with 1 M H_2_SO_4_ (100 °C for 2.5 h) was hydrolysed. Thus, the cellulose content could be estimated by the difference in glucose obtained by Saeman hydrolysis and this milder hydrolysis method. Then, all the hydrolysed samples were neutralized with ammonia and reduced using NaBH_4_. Next, the neutral sugars present in the samples were derivatized as their corresponding alditol acetates using acetic anhydride and 1-methylimidazole as a catalyst. Finally, the alditol acetates were isothermally separated at 220 °C by gas chromatography (Perking Elmer-Clarus 400 (CR1)), using a 30 m column DB-225 (J&W Scientific, Folsom, CA, USA) with an internal diameter of 0.25 µm and a film thickness of 0.15 µm, and analysed with an FID detector. Standard mixtures of neutral sugars were derivatized routinely to check GC response factors. Uronic acids were determined colourimetrically, as total uronic acids [29], using a sample hydrolysed for 1 h at 100 °C in 1 M H_2_SO_4_ [4]. Representative GC-chromatograms of carbohydrate analysis for different *Aloe vera* samples after Saeman hydrolysis can be found in the Figure S1 of the Supplementary Materials.

### 2.5. Degree of Acetylation of Acemannan by Nuclear Magnetic Resonance (NMR) Analysis

The degree of acetylation of the acemannan polymer, as the main component of AIR-samples, was analysed using the method described by Bozzi et al. (2007) [30]. Therefore, 2 mg of hydrolysed sample together with 2 mg nicotinamide (Sigma-Aldrich, St. Louis, MO, USA) were dissolved in heavy water (Sigma-Aldrich, St. Louis, MO, USA); nicotinamide was used as an internal shift standard, because its peaks appear after 7.5 ppm, which are well-separated from other *Aloe vera* derived proton peaks [31]. Then, the solubilized samples were transferred into Wildman Economic 5-mm NMR tubes. The ^1^H-NMR spectra at 300.13 MHz were recorded on an Avance 300 spectrometer (Bruker, Billerica, MA, USA), equipped with a 5-mm broadband multinuclear z-gradient (BBO) probe head. The area under the curve (AUC) was calculated using the acetyl group signal corresponding to nicotinamide. The relative degree of acetylation of processed samples in relation to the fresh (reference) sample was calculated using the following Equation:

Relative degree of acetylation = (AUC processed/AUC reference) × 100

Representative ^1^H-NMR-spectra from different *Aloe vera* samples to determine the degree of acetylation of acemannan can be found in the Figures S2 and S3 of the Supplementary Materials.

### 2.6. Culture Conditions

The human intestinal cell line HT-29 (#HTB-38, ATCC, Manassas, VA, USA) was seeded with 4 × 10^4^ cells/cm^2^ in growth medium (DMEM medium (ATCC), supplemented with 10% FBS (Gibco, Carlsbad, CA, USA)) in 12-well microplates (when necessary round cover slides have been added into the wells before) and incubated overnight at 37 °C, 4% CO_2_ and 95% RH for attachment. For the experiments, at a confluence of ~80% growth medium was changed by sample-containing growth medium. The samples (pasteurized, dehydrated and AIR) were dissolved in concentration of 30 g/L (3×), 10 g/L (1×), 3 g/L (0.3×), 1 g/L (0.1×) in culture medium; the pH was adjusted to pH 7.3 with NaOH, and to inhibit bacterial growth, 50 µg/mL of gentamycin (Sigma-Aldrich, St. Louis, MO, USA) were added. The commercial samples (200× ILF and ILL) have been dissolved with 15 g/L (3×), 5 g/L (1×), 1.5 g/L (0.3×), 0.5 g/L (0.1×) in growth medium; otherwise, the treatment was the same as for the other samples. As growth control, cells were just grown in growth medium under standard conditions; for the positive control for necrosis, H_2_O_2_ to a final concentration of 20 mm was added to the cells.

### 2.7. Cell Viability and Necrosis

To measure the impact of the samples on the metabolic activity, the cells were incubated for 24 h under the above-mentioned conditions. Then the sample-containing medium was removed, the cells were washed twice with PBS and 1 mL growth medium, containing 20 µL/mL 2-(4-iodophenyl)-3-(4-nitrophenyl)-5-(2,4-disulfophenyl)-2H-tetrazolium (WST-1; Clontech, Mountain View, CA, USA), was added. Afterwards, the cells were incubated further for 4 h.

Finally, for quantitative analysis, 800 µL WST-1 containing growth medium was centrifuged (1 min, 8000× *g*) to precipitate possible cell debris before measuring the absorbance at λ = 440 nm and λ = 690 nm (as background) (Mecasys Optizen-Pop, Daejeon, South Korea). The absorbance of the growth control was normalized to 100%, all other values are expressed relative to this (relative WST-activity).

To detect necrosis, 0.05% (final concentration) trypan blue (Biowest, Kansas City, MO, USA) was added, incubated for 5 to 10 min, the cells grown on cover slides were washed with PBS and then observed under the microscope (Axioskop 40 FL, Zeiss, Oberkochen, Germany) [25].

### 2.8. Statistical Analysis

Values for carbohydrate composition, monosaccharide composition and degree of acetylation are expressed as mean ± standard deviation (SD), cytotoxicity values are expressed as mean ± 1.96 × standard error of mean (1.96 × SEM). To calculate SEM for relative WST-activity the following repetitions have been done: 5 × DAG60, 3 × DAG80, 3 × DAG100, 4 × PAG60, 4 × PAG80, 4 × PAG100, 3 × AAG60, 4 × AAG80, 3 × AAG100, 4 × ILF, 4 × AILF, 3 × ILL, 8 × H_2_O_2_. The parameter CC_50_ was estimated by interpolating the half-maximal relative cell viability in the Boltzmann equation fitted to the experimental plot of relative cell viability vs. concentration using the software Origin 5.0 (Microcal Software, Northampton, MA, USA). Statistical analysis was performed by one-way ANOVA followed by Tukey’s post-hoc test to determine significant difference (*p* < 0.05) between multiple means using the software Statistica 13.3 (StatSoft, Hamburg, Germany).

## 3. Results

### 3.1. Molecular Identification

The commercial Aloe powder ILF as well as the plants, previously visually identified as *Aloe vera*, were authenticated by PCR amplification and DNA sequencing of regions of *rbcL* and *matK* plastid genes, which have been proposed as the core plant barcode [23]. The DNA sequences obtained were of 557 bases for the *rbcL* fragment and of 851 bases for *matK* in the fresh and industrial (ILF) sample. For similarity searches, these sequences were submitted to the Barcode of Life Data Systems (http://www.boldsystems.org/ (accessed on 1 June 2021)) using the *Plant Identification* tool and all fragments displayed 100% of similarity (without gaps) with the corresponding regions of *Aloe vera*, confirming the identity of the plant material. A table with a similarity index and a phylogenetic tree can be found in the Supplementary Materials (Table S5 and Figure S4, respectively). The sequences were deposited in GenBank under the accession numbers MW176074 (*rbcL*) and MW176075 (*matK*).

### 3.2. Analysis of Carbohydrate Composition

**Figure 1:**
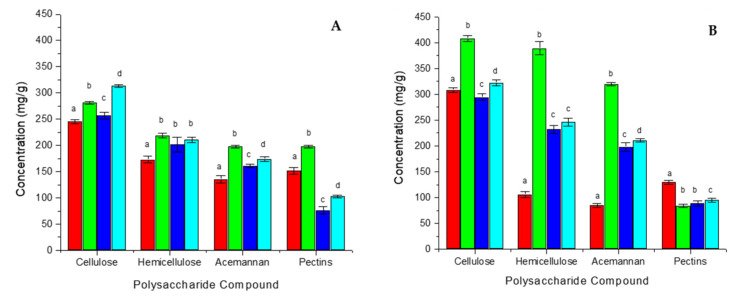
Polysaccharide compounds from the different *Aloe vera* gel samples: ILF (red), DAG60 (green), DAG80 (blue), DAG100 (cyan) (**A**). AILF (red), AAG60 (green), AAG80 (blue), AAG100 (cyan) (**B**). Error bars indicate SD. Significant differences for different samples for each compound are indicated by different letters. (The exact values can be found in Table S1 of the Supplementary Materials).

The major polymeric constituents from the dehydrated (DAG60, 80, 100) and commercial (ILF, ILL) samples and their respective alcohol insoluble residues (AAG60, 80, 100, AILF) were determined (Figure 1). When AIRs were extracted from the dehydrated and commercial samples, all exhibited a high viscosity, which indicates the presence of insoluble components. The sample ILL was completely soluble, which means that no AIR was obtained, and was therefore excluded from this comparison (values can be found in the Tables S3 and S4 of the Supplementary Materials).

The relative polysaccharide composition between the dehydrated samples (DAG60, 80, 100) is quite similar with ~35–39% cellulose, ~26–29% hemicelluloses, ~22–25% acemannan and ~11–13% pectins (Figure 1A) and does not differ much from the respective AIRs (~33–36% cellulose, ~28–32% hemicelluloses, ~24–26% acemannan and ~7–11% pectins) (Figure 1B); although the absolute values for cellulose, hemicellulose and acemannan in the sample AAG60 are significantly higher (Figure 1B). In contrast, the relative composition of the industrial sample ILF differs from the dehydrated samples with a lower hemicellulose (~25%) and acemannan (~19%) and a higher pectin (~21%) content. The AIR from ILF has a much higher content of cellulose (~50%) and pectins (~21%) and consequently a lower content of hemicelluloses (~17%) and acemannan (~14%) than the AIRs from the dehydrated samples (Figure 1B). Acemannan is the most interesting polysaccharide of the samples as it contributes to the physiological effects. Thus, it is important to note that the industrial sample ILF, as well as its respective AIR (AILF), has a significant lower acemannan content than the laboratory samples DAG and AAG, respectively (Figure 1). Another trend is easier to observe in the polysaccharide enriched AIR-fraction: as expected, an increase of the drying temperature caused an increase of polysaccharide degradation—as can be seen for the cellulose, hemicellulose, and acemannan composition of AAG60 vs. AAG80, and AAG100 (Figure 1B), respectively. But astonishingly, the cellulose and acemannan content for AAG80 were significantly lower than for the AAG100 sample (Figure 1B).

**Figure 2:**
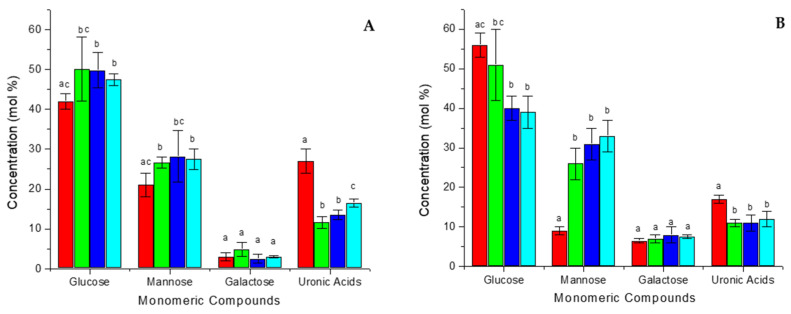
Monosaccharide composition of polysaccharides isolated from the different *Aloe vera* gel samples: ILF (red), DAG60 (green), DAG80 (blue), DAG100 (cyan) (**A**). AILF (red), AAG60 (green), AAG80 (blue), AAG100 (cyan) (**B**). Error bars indicate SD. Significant differences for different samples for each compound are indicated by different letters. (The exact values can be found in Table S2 of the Supplementary Materials).

Additionally, the monosaccharide composition, i.e., glucose, mannose, galactose and uronic acids, of the major components was quantified from the dehydrated (DAG60, 80, 100) and commercial (ILF) samples as well as their respective alcohol-insoluble residues (AAG60, 80, 100, AILF) (Figure 2).

The glucose content does not vary among the dehydrated samples (~48–50%) (Figure 2A), but diminishes with increasing drying temperatures for the respective AIRs (50.6 to 38.6%) (Figure 2B). Inversely, the mannose content increases with increasing drying temperature for AIRs (26.4 to 32.8%) (Figure 2B), while no such trend can be observed for the dehydrated samples (~27–28%) (Figure 2A). Again, the industrial ILF sample differs from the dehydrated samples: the glucose and mannose contents are lower (41.6% and 20.5%), while uronic acids are more present (27.0%) (Figure 2A), which correlates with the higher pectin content (Figure 1A). AILF has a much higher glucose (55.8%) and much lower mannose (8.6%) content, and the relative uronic acid content (16.8%) decreases, too (Figure 2B). This is consistent with an increase of cellulose and a decrease in hemicelluloses and acemannan for AILF compared to ILF. The high glucose content in all samples can be explained mainly by the presence of cellulose (Glc-β1 → 4-Glc) and also acemannan. The second most abundant carbohydrate is mannose, the main constituent of acemannan. The concentration of uronic acids correlates with the concentration of pectins, while galactose is principally a minor constituent of acemannan.

### 3.3. Cytotoxicity

**Figure 3:**
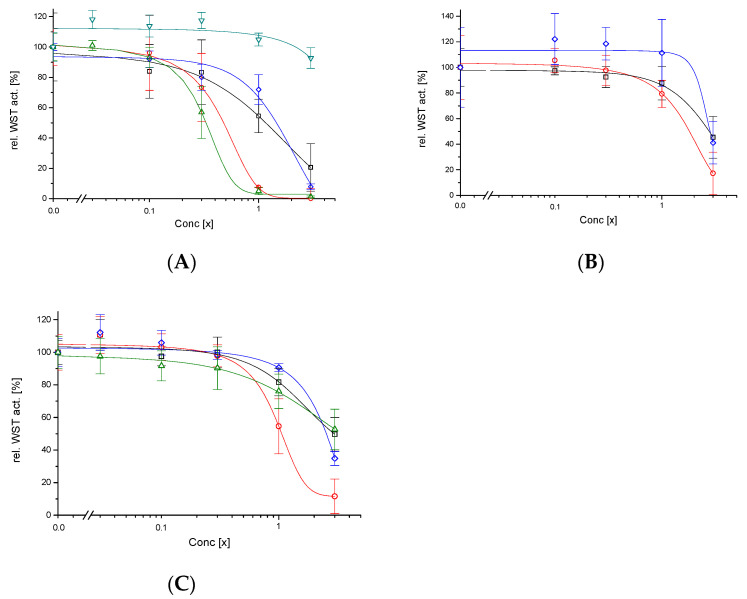
Estimation of cytotoxicity by measuring metabolic activity of HT-29 after being exposed for 24 h to differently treated Aloe fillets: dehydrated DAG (**A**), pasteurized PAG (**B**), AIRs AAG (**C**). Samples treated at 60 °C (black square), 80 °C (red circle), 100 °C (blue diamond), ILF (green up-triangle) or ILL (cyan down-triangle). The growth control (0×) was set to 100%, the value of the positive control (20 mm H_2_O_2_) with 0.24 ± 0.96 is not shown. Error bars indicate SEM × 1.96.

The effect of different heating procedures on *Aloe vera* gel was evaluated by estimating their cytotoxic effects. This was determined by the reduction of tetrazolium salt WST by dehydrogenase activity of the human intestinal cell line HT-29, which represents a cell line of first contact when those products would be ingested. First, the dehydrated Aloe fillets (DAG60, 80, 100, ILF) exhibited a low cytotoxicity for DAG60 with CC_50_ = 1.1× and for DAG100 with CC_50_ = 1.5×. Not expected was a higher cytotoxicity for DAG80 with CC_50_ = 0.4× (Figure 3A). The concentration of 1× refers to 10 g/L (respective 5 g/L for 200× concentrated powders) and is the recommended concentration for foodstuff. The control ILL exhibited up to 3× (over)concentrated no cytotoxic effect on HT-29 (Figure 3A); due to the low polysaccharide content this sample was probably adulterated—in this case at least the adulterants have not been cytotoxic. In contrast, the industrial sample ILF, which has been dehydrated by belt dry at temperatures between 70–80 °C, was with a CC_50_ = 0.3× cytotoxic for HT-29. To distinguish whether the heat itself or its combination with the dryness caused the cytotoxic effect of the DAG80 sample, the Aloe fillets were additionally heated under humid conditions (PAG60, 80, 100): In all cases cytotoxicity decreased, for PAG60 to CC_50_ = 2.8×, for PAG80 to CC_50_ = 1.6× and for PAG100 to CC_50_ = 2.5× (Figure 3B). This behaviour, a peak cytotoxicity at the intermediate temperature (PAG80), may be explained by two factors acting in opposite directions: one factor increases cytotoxicity with increasing temperature, while the other factor, independently, decreases cytotoxicity with increasing temperature.

One of these factors may be the polysaccharides and/or modifications of them due to the heat treatment. Therefore, and to our knowledge for the first time, the cytotoxicity of Aloe fillet AIRs, a fraction enriched with polysaccharides, was also determined (AAG60, 80, 100, AILF). Here, too, cytotoxicity was reduced in all cases compared to the dehydrated samples: for AAG60 to CC_50_ = 2.9×, for AAG80 to CC_50_ = 0.9×, for AAG100 to CC_50_ = 2.7× and for AILF to CC_50_ > 3× (Figure 3C).

**Figure 4:**
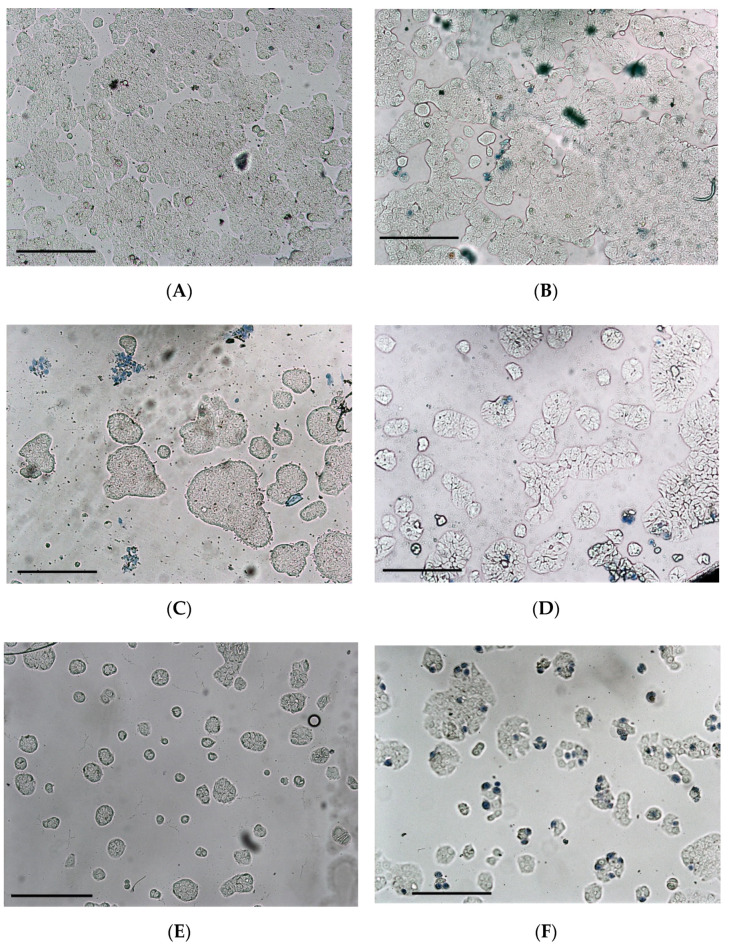

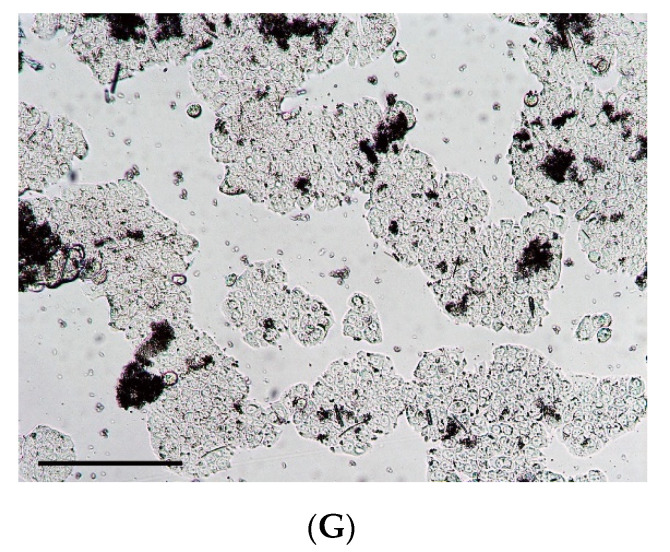
Trypan blue staining of HT-29 cells exposed for 24 h with different Aloe-samples: Control (**A**), 1× DAG60 (**B**), 0.3× DAG80 (**C**), 1× PAG80 (**D**), 1× AAG80 (**E**), 0.3× ILF (**F**) and 3× AILF (**G**). Initial confluence was ~80% in all cases; frequently not many blue stained (dead) cells can be seen as those are washed away in a previous washing step (confluence can be evaluated). The bar indicates 50 µm.

In addition to the metabolic activity (WST test), the cytotoxicity was confirmed by trypan blue staining. The Aloe samples treated at 60 °C or 100 °C, which have CC_50_ > 1×, do not exhibit cytotoxic effects up to a concentration of 1× (Figure 4B). This is in contrast to the samples treated at 80 °C: The dehydrated sample (DAG80) reduced cell growth already at 0.3× (Figure 4C); the corresponding AIR (AAG80) shows a clear cell growth inhibition at 0.9× (Figure 4E), while the pasteurized sample PAG80 only slightly inhibited cell growth at 1×, but morphological changes can be observed (irregular protuberance) (Figure 4D). In general, only few blue-stained cells can be seen because after the incubation with the sample, the medium was withdrawn, the cells were washed with PBS and fresh medium was added for the WST test, which was done in parallel; in this washing step most of the dead (blue stained) cells were washed away. The sample ILF exhibited a stronger cytotoxic effect on HT-29 at 0.3× (Figure 4F), similar to that previously reported on HeLa and Caco-2 [5,22], but the AIRs of ILF (AILF) showed a very low cytotoxicity (Figure 4G).

### 3.4. Polysaccharide Modifications

Finally, the polysaccharide modifications introduced by the heat treatment of the samples were analysed. The degree of acetylation was determined by NMR. Since the dehydrated samples (DAG, ILF) could not be dissolved completely in D_2_O as required, the degree of acetylation could only be determined for their respective AIRs (AAG, AILF). Freshly lyophilized Aloe gel was used as a reference. Generally, the AIR-fraction is enriched in acemannan, compared to the dehydrated samples—with the exception of the industrial ILF-sample (Figure 1). This is consistent with the higher content of mannose and galactose in the AIR fraction compared to the dehydrated samples, and again with the exception of the ILF sample (Figure 2). AAG60 has the highest increase in acemannan content; samples treated at higher temperatures (AAG80, 100, AILF) exhibited lower increases. Different chemical modifications may occur at different temperatures; it is known that debranching reactions start at temperatures >60 °C, especially for terminal galactose. At temperatures >70 °C deacetylation reactions begin and polymers may gain molecular weight through other reactions [13]. AAG60, which derive from the DAG60, showed with 14.0%, by far, the lowest percentage of deacetylation. All other samples (AAG80, 100, AILF) that were treated at higher temperatures exhibited, in consequence, with values ranged from 46.5 to 65.1%, a much higher deacetylation (Table 1). The sample ILL has an apparently high percentage of deacetylation, but it has to be considered that the absolute acemannan content was, probably due to adulteration, very low (Table 1).

## 4. Discussion

Polysaccharides are the most abundant components in vegetal tissues. Generally, they can be divided into cell wall polysaccharides, such as cellulose, hemicelluloses and pectins, and storage polysaccharides like acemannan, in the case of *Aloe vera* [8]. Acemannan is considered a bioactive compound, despite of not being a secondary metabolite. Therefore, it is desired that commercial products from *Aloe vera* have a high content of polysaccharides, in particular acemannan, and this is also the main reason why different effects of drying procedures –including method, temperature, pH, O_2_ and the presence of other compounds—as well as the polysaccharides and their chemical and biological properties—like chain length, monosaccharide composition, emulsifying activity or structural conformation– are widely studied [32].

Although acemannan is due to its bioactivity the most interesting polysaccharide in *Aloe vera* gel, the industrial sample ILF, which is especially prepared in order to keep a high fiber content [22], has with 19.2 mol% the lowest acemannan content compared to the other DAG-samples (~22–25 mol%) (Figure 1A). Moreover, when simulating the industrial process used to produce ILF (belt drying at ~80 °C), the laboratory sample suffered a similar effect: it had the lowest acemannan content (among all DAG samples), which was even more pronounced in the polysaccharide-enriched AIR-fraction AAG (Figure 1B).

Despite a similar composition of the dehydrated samples, their cytotoxic effect on the human intestine cell line HT-29 was quite different. When dehydrated, either at 60 °C or 100 °C, the samples were not cytotoxic (DAG60 CC_50_ = 1.1× and DAG100 CC_50_ = 1.5×), but, astonishingly, when dehydrated at 80 °C (DAG80) the sample was, with CC_50_ = 0.4×, rather cytotoxic—similar to the industrial sample ILF, which has a CC_50_ = 0.3×. This cytotoxic effect cannot be associated directly with the acemannan concentration of the samples; for example, DAG80 with 161 mg/g or ILF with 135 mg/g were cytotoxic, while DAG100 with 174 mg/g or AILF with 85 mg/g were not (Figure 1 and Table 1). In general, the polysaccharide-enriched AIR-samples were less cytotoxic than the dehydrated samples, indicating that the polysaccharides were not the cytotoxic principle.

Applying heat during the drying procedure can be associated to several effects: First, polysaccharides and proteins may covalently bond via Maillard reactions, especially under hot and dry conditions, and can easily be seen as an increased browning of the final product, which will have altered biological activities [33]. Applying humid (PAG) instead of dry (DAG) heat reduces Maillard reactions, as evidenced by less browning of the final product [13,34], and a two- to three-fold reduction of cytotoxicity to the HT-29 human intestinal cell line (Table 1).

Another possibility is that the heat may promote deacetylation reactions of the acemannan polymer. In fact, Chokboribal et al. (2015) [17] described that the loss of acetyl groups from acemannan, which readily occurs within 5 min at 80 °C under alkaline conditions, leads to a reorganized topology through the formation of new intra- and intermolecular hydrogen bonds. Possibly due to steric hindrance, deacetylation increased hydrophobicity and crystallinity. Compared to a freshly lyophilized Aloe gel, with a normalized degree of acemannan acetylation of 100%, only AAG60 showed with 14% a low percentage of deacetylation; on the contrary, at temperatures of 80–100 °C the percentage of deacetylation increased drastically to 46–58% and reached the highest value for the industrial sample AILF with 65%. This confirms several studies indicating that structural changes in polysaccharides, and in particular of acemannan, like deacetylation and loss of galactose side chains thus having a lower degree of branching, are highly dependent on the temperature [11,12,13] and that these may promote a considerable reduction of their biological activity [17,19,35]. Here, it is reported for the first time that, despite these changes, the cytotoxicity of AIR samples is, with CC_50_ = 0.9–3×, lower than the cytotoxicity exhibited by the dehydrated samples. Also, Chokboribal et al. (2015) [17] reported that solutions containing up to 4 g/L of deacetylated acemannan did not exhibit cytotoxic effects on gingival fibroblasts.

On the other hand, applying heat has also advantages: high temperatures are a cost-effective manner to accelerate the drying process and, at the same time, to remove the toxic aloin, which is by regulations limited to 10 ppm in foodstuff [36]. Chang et al. (2006) [11] showed that after heating of a sample at 60 °C during 1 h, the concentration of the glycosylated anthrone aloin A remained nearly constant while at 80 °C the concentration was reduced by nearly one third; the authors demonstrated that only a small part of aloin A was converted to the non-glycosylated anthraquinone aloe-emodin but most of it to several other, still unknown, substances. Additionally, heating to higher temperatures also reduces the number of pathogenic microorganisms and thus increases the shelf life of a product.

Respective to the cytotoxicity, two temperature-dependent but contrary processes peaked at 80 °C: (i) the reduction of toxicity with increasing temperature due to the elimination of aloin [37], and (ii) the introduction of a toxic principle by chemical reaction under dry heat conditions. It can be excluded that the toxic principle is an altered polysaccharide (e.g., deacetylated acemannan), because the polysaccharide-enriched AIR fraction (AAG) was about two times less cytotoxic than the corresponding DAG samples. On the other hand, heat-induced modifications of aloin A indicates the complexity of reactions that can suffer secondary metabolites. This means that the toxic principle is probably a low molecular weight substance that is sufficiently hydrophilic to be washed away with 85% ethanol.

## 5. Conclusions

When processing Aloe gel, their heating above 60 °C should be avoided in order (i) to not introduce a toxic principle of low molecular weight (although it can be removed, e.g., by ethanolic precipitation or by treatment with charcoal [22]), and (ii) to not introduce structural modifications of acemannan (in particular deacetylation), which would reduce its biological activity.

## Figures and Tables

**Table 1:** Degree of acetylation of acemannan from AIRs dehydrated at different temperatures compared to freshly lyophilized Aloe gel. Mean ± SD. Comparison of median cytotoxic concentration on HT-29 cells according the sample preparation.

Samples	Degree of Deacetylation	Deacetylation [%]	Cytotoxicity (CC_50_)		
			Dehydrated	AIR	Pasteurized
Lyophilized Aloe gel	0.43 ± 0.01	0.0	-	-	-
Aloe Gel 60	0.37 ± 0.01	14.0	1.1×	2.9×	2.8×
Aloe Gel 80	0.18 ± 0.01	58.1	0.4×	0.9×	1.6×
Aloe Gel 100	0.23 ± 0.01	46.5	1.5×	2.7×	2.5×
ILF	0.15 ± 0.01	65.1	0.3×	>3×	-
ILL	0.08 ± 0.01	81.4	>3×	-	-

## Data Availability

The data presented in this study are available on request from the corresponding author.

## Supplementary Materials

The following are available online at https://www.mdpi.com/article/10.3390/foods11050745/s1 Figure S1: Representative GC-chromatograms after Saeman hydrolysis for the different *Aloe vera* samples: DAG60, DAG80, DAG100, ILF; Figure S2: ^1^H-NMR spectra from fresh *Aloe vera* gel, DAG60, DAG80 and DAG100 samples to determine the degree of acetylation of acemannan; Figure S3: Comparison of range spanning 4.4–1.7 ppm chemical shift of ^1^H-NMR spectra from DAG60, DAG80 and DAG100; Figure S4: A taxon identification tree for *Aloe vera* and related species created using the UPGMA analysis of Jukes-Cantor nucleotide substitution model based on combined *rbcL* and *matK* sequences. Bootstrap values are shown next to the nodes; Table S1: Polysaccharides isolated from dehydrated and commercial *Aloe vera* samples and their respective AIRs; Table S2: Monosaccharide composition of polysaccharides isolated from the industrial *Aloe vera* gel sample ILL and the respective AIR; Table S3: Polysaccharides isolated from the industrial *Aloe vera* gel sample ILL; Table S4: Monosaccharide composition of polysaccharides isolated from the industrial *Aloe vera* gel sample ILL; Table S5: Sequence similarities for the two barcode genes *rbcL* and *matK* of the analysed samples with different Aloe species. Sequence similarity was 100% with *Aloe vera*.

## Abbreviations

AAG	AIR-fraction from DAG
AILF	AIR-fraction from ILF
AIR	Alcohol Insoluble Residue
CC_50_	Median Cytotoxic Concentration
D_2_O	Heavy water
DAG	Dehydrated *Aloe vera* Gel
ILF	*Aloe vera* Inner Leaf gel, high Fiber content
PAG	Pasteurized *Aloe vera* Gel
WST	Water-Soluble Tetrazolium salt (2-(4-iodophenyl)-3-(4-nitrophenyl)-5-(2,4-disulfo-phenyl)-2H-tetrazolium)
