# Supplementary material for: Dry but Not Humid Thermal Processing of Aloe vera Gel Promotes Cytotoxicity on Human Intestinal Cells HT-29
Source: Foods. 2022 Mar 3;11(5):745. doi: 10.3390/foods11050745 (PMC8909460; doi:10.3390/foods11050745)
Supplement: Supplementary file 1 [file foods-11-00745-s001.zip › foods-1574744-supplementary.pdf]

Supplementary Materials

Dry but Not Humid Thermal Processing of *Aloe vera* Gel Promotes Cytotoxicity on Human Intestinal Cells HT-29

Zaira López, Michelle N. Salazar Zúñiga, Antoni Femenia, Gustavo J. Acevedo-Hernández, Jaime A. Godínez Flores, M. Eduardo Cano & Peter Knauth*

* Corresponding author: Peter Knauth knauth@gmx.de

Phytochemical Characterization

The focus of this work is to evaluate the impact of the drying conditions of Aloe fillet or Aloe gel on the polysaccharide constitution and the resulting cytological effects. Thus previously published results, focussing on an extensive phytochemical characterization, are not repeated. Kahramanoglu *et al.* (2019) published a review on the secondary metabolites found in *Aloe vera* plants [1]. The chemical composition of Aloe skin, Aloe fillet, Aloe gel and their respective AIRs have been published previously by Femenia *et al.* (1999) [2]. Moreover, the main chemical structures of pectic polysaccharides and acemannan present in the Aloe plant have also been published by Minjares-Fuentes *et al.* (2018) and Chokboribal *et al.* (2015), respectively [3,4]. In this paper, the chemical composition of the main polysaccharides present in the *Aloe vera* samples were characterized using gas chromatography and the degree of acetylation of acemannan by NMR analysis.

Representative GC-chromatograms of carbohydrate analysis of *Aloe vera* samples after Saeman hydrolysis:

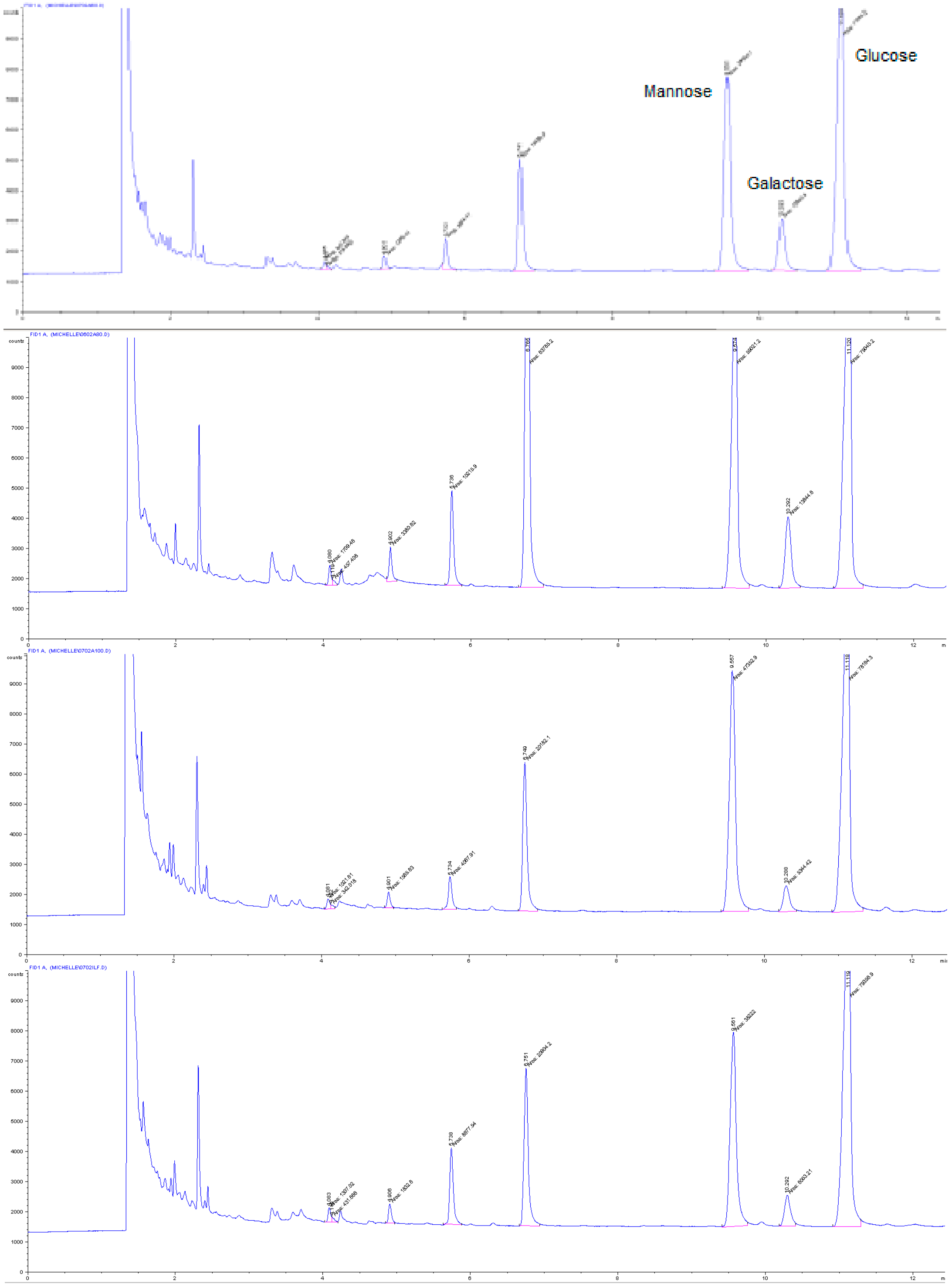

DAG60

DAG80

DAG100

ILF

Figure S1: Representative GC-chromatograms after Saeman hydrolysis for the different *Aloe vera* samples: DAG60, DAG80, DAG100, ILF

The concentration of sugars (Tab. 2) were obtained from area of GC-FID peaks (Fig. 1) and the polysaccharides contents (Tab. 1) by estimation from the differences of each monosaccharide obtained by Saeman hydrolysis.

Table S1: Polysaccharides isolated from dehydrated and commercial *Aloe vera* samples and their respective AIRs. Mean ± SD.

Sample	Cellulose [mg/g]	Hemicelluloses [mg/g]	Acemannan [mg/g]	Pectins [mg/g]
DAG60	281.1 ± 2.4	218.8 ± 4.5	198.0 ± 7.1	97.7 ± 2.7
DAG80	256.9 ± 6.9	201.5 ± 13.6	161.0 ± 3.7	76.2 ± 6.9
DAG100	313.3 ± 2.3	210.8 ± 5.0	174.1 ± 4.5	102.6 ± 2.1
ILF	245.3 ± 4.3	173.1 ± 6.0	135.6 ± 6.9	151.4 ± 6.1
AAG60	408.2 ± 6.3	389.4 ± 13.2	319.4 ± 3.5	83.8 ± 6.5
AAG80	294.2 ± 6.4	232.5 ± 7.8	198.3 ± 8.3	88.7 ± 5.7
AAG100	322.5 ± 5.5	246.1 ± 8.1	210.8 ± 3.3	95.0 ± 3.5
AILF	308.3 ± 4.6	106.0 ± 5.3	85.3 ± 3.8	129.9 ± 4.1

Table S2: Monosaccharide composition of polysaccharides isolated from dehydrated and commercial *Aloe vera* samples and their respective AIR. Mean ± SD.

Sample	Glucose [mol%]	Mannose [mol%]	Galacose [mol%]	Uronic acids [mol%]
DAG60	50.1 ± 8.0	26.6 ±1.4	4.9 ± 1.7	11.6 ± 1.5
DAG80	49.8 ± 4.4	28.2 ± 6.5	2.5 ± 1.1	13.5 ± 1.2
DAG100	47.5 ± 1.5	27.5 ± 2.6	3.0 ± 0.3	16.5 ± 1.1
ILF	41.6 ± 2.3	20.5 ± 2.9	3.2 ± 1.2	27.0 ± 3.3
AAG60	50.6 ± 9.2	26.4 ± 4.2	7.0 ± 1.0	10.9 ± 1.2
AAG80	40.3 ± 2.6	31.3 ± 4.1	7.6 ± 2.3	11.1 ± 1.9
AAG100	38.6 ± 4.3	32.8 ± 4.4	7.5 ± 0.5	12.1 ± 1.8
AILF	55.8 ± 3.2	8.6 ± 1.0	6.5 ± 0.6	16.8 ± 1.2

Representative ^{1}H-NMR-spectra from different *Aloe vera* samples to determine the Degree of Acetylation of Acemannan.

PDF Complete
Your complimentary use period has ended. Thank you for using PDF Complete.
Click Here to upgrade to Unlimited Pages and Expanded Features

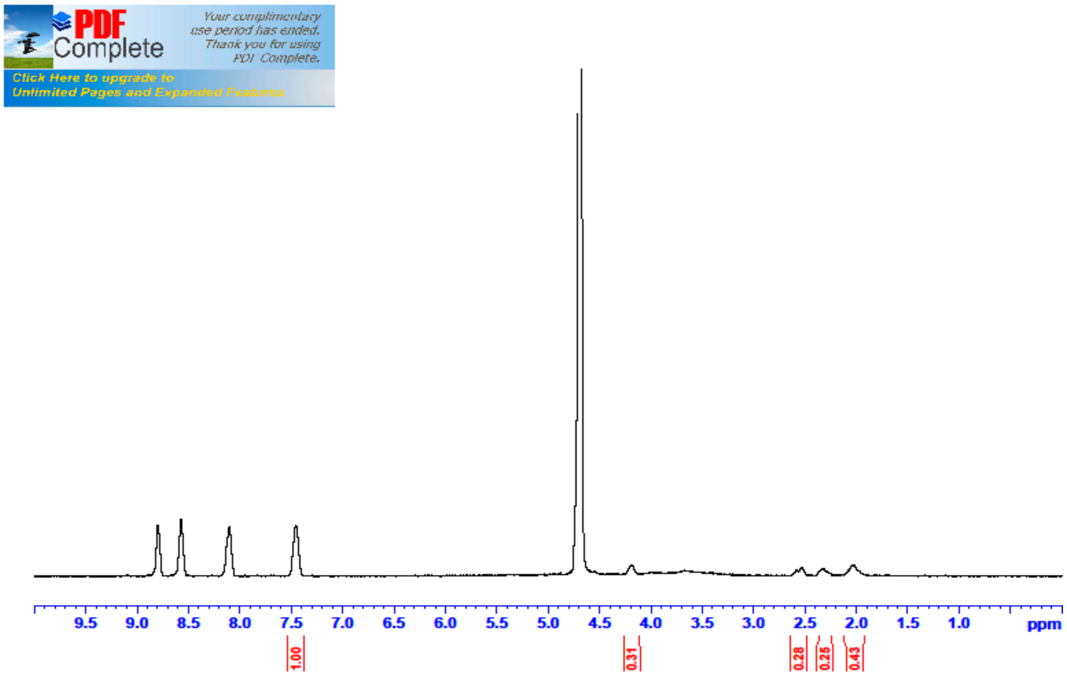

fresh

Aloe vera

PDF Complete
Your complimentary use period has ended. Thank you for using PDF Complete.
Click Here to upgrade to Unlimited Pages and Expanded Features

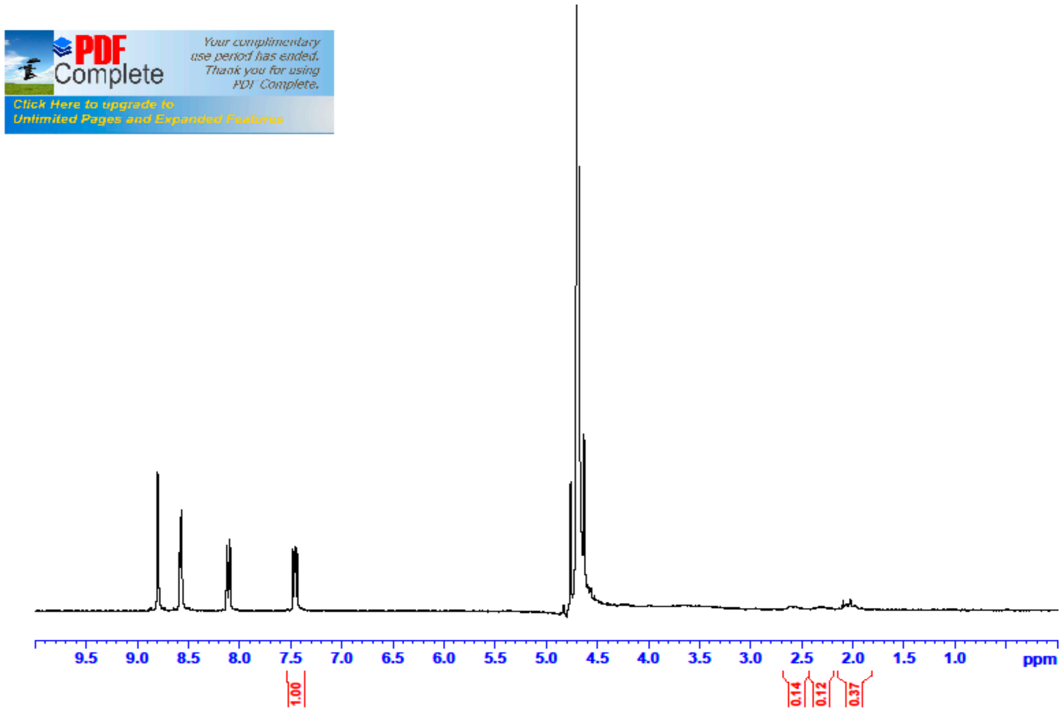

DAG60

PDF Complete
Your complimentary use period has ended. Thank you for using PDF Complete.
Click Here to upgrade to Unlimited Pages and Expanded Features

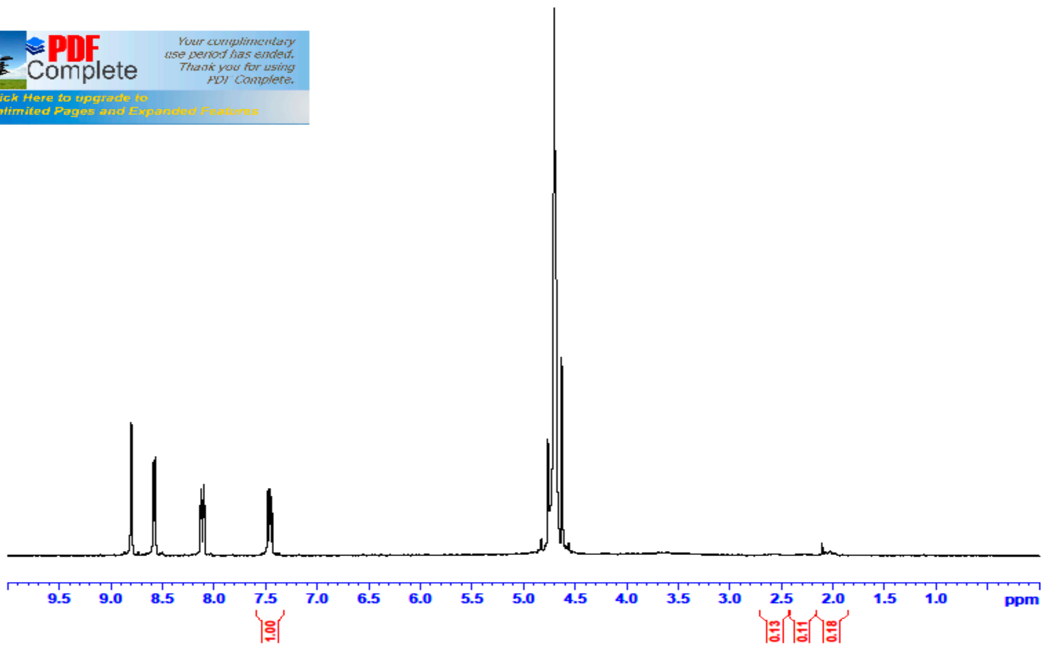

DAG80

PDF Complete
Your complimentary use period has ended. Thank you for using PDF Complete.
Click Here to upgrade to Unlimited Pages and Expanded Features

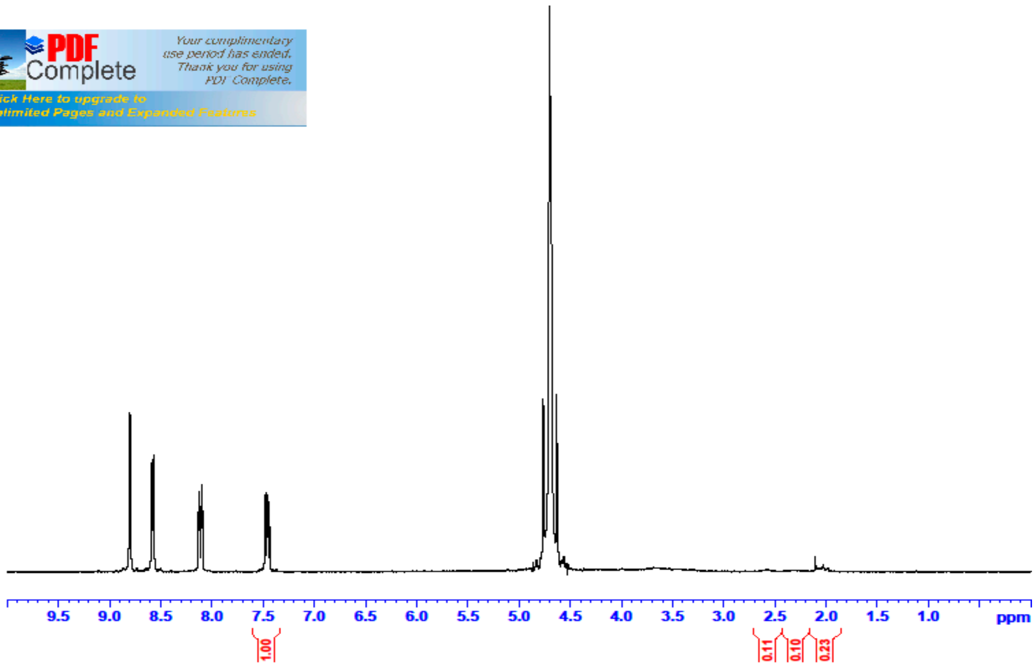

DAG100

Figure S2: ^{1}H-NMR spectra from fresh *Aloe vera* gel, DAG60, DAG80 and DAG100 samples.

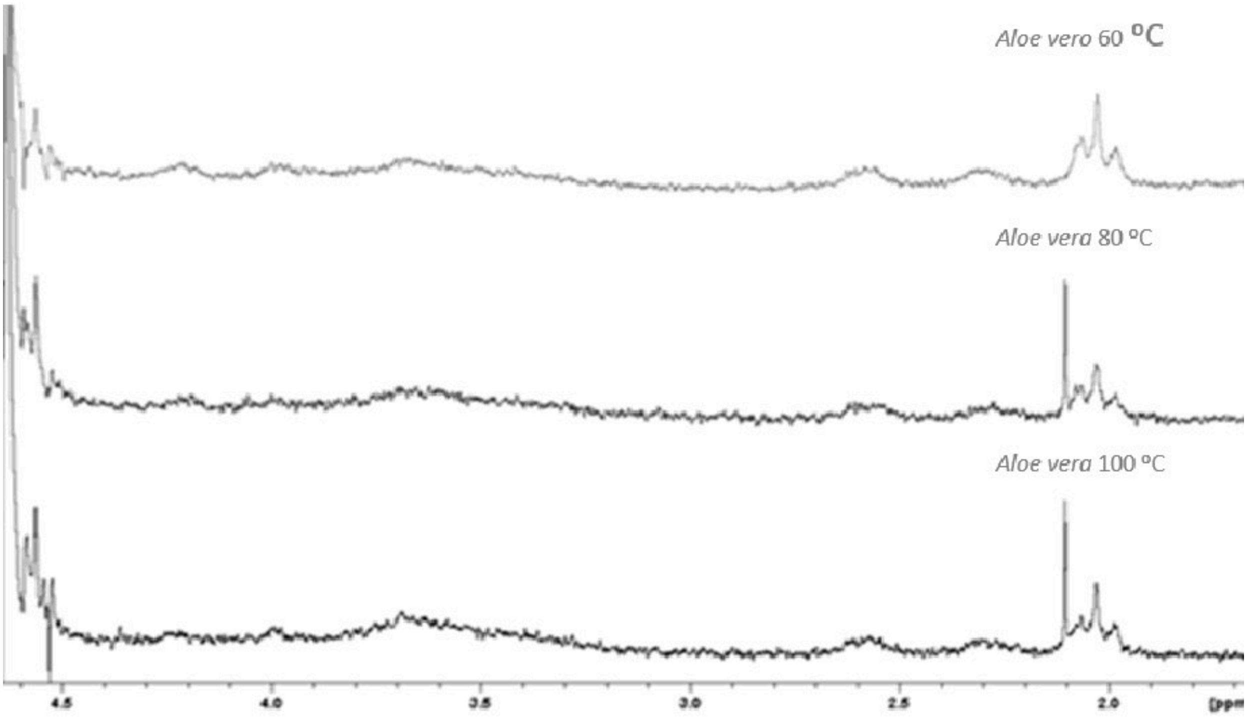

Figure S3: Comparison of range spanning 4.4 – 1.7 ppm chemical shift of ^{1}H-NMR spectra from DAG60, DAG80 and DAG100. Peaks at ~2.0 ppm represent acemannan. The peak at 1.9 ppm indicates the modifications introduced by the heat for the samples DAG80 and DAG100.

The second industrial sample, ILL, has a similar relative constitution of polysaccharides than the other samples; nevertheless, its absolute polysaccharide content was about 2.7 times lower (Tab. 3). Probably this product was adulterated, maybe with maltodextrin, a common adulterant for Aloe powders [5]. This would also explain its relatively high glucose content (Tab. 4) and that AIR could not be prepared.

Table S3: Polysaccharides isolated from the industrial *Aloe vera* gel sample ILL. Mean ± SD.

Sample	Cellulose [mg/g]	Hemicelluloses [mg/g]	Acemannan [mg/g]	Pectins [mg/g]
ILL	79.2 ± 5.2	81.7 ± 5.1	66.2 ± 11.4	53.0 ± 8.7
AILL	--	--	--	--

Table S4: Monosaccharide composition of polysaccharides isolated from the industrial *Aloe vera* gel sample ILL. Mean ± SD.

Sample	Glucose [mol%]	Mannose [mol%]	Galacose [mol%]	Uronic acids [mol%]
ILL	56.9 ± 2.7	18.4 ± 1.3	2.8 ± 1.6	15.1 ± 4.9
AILL	--	--	--	--

Molecular Characterization

The sequences for the DNA barcode genes *rbcL* and *matK* for the fresh tissue and industrial sample ILF are identical. The industrial sample ILL was adulterated (probably with maltodextrin) and therefore not considered as an adequate control; this sample was not sequenced. The sequences were deposited in GenBank under the accession numbers MW176074 (*rbcL*) and MW176075 (*matK*).

Table S5: Sequence similarities for the two barcode genes *rbcL* and *matK* of the analysed samples with different *Aloe* species. Sequence similarity was 100% with *Aloe vera*.

Plant sample	DNA barcode gene	Best matches*	GenBank ID	Sequence cover (%)	Sequence similarity (%)
Aloe Dry Powder & Aloe Fresh Tissue (sequences are identical)	*rbcL*	*Aloe vera*	JQ273907	99.64	100.00
		Aloe purpurea	Z73690	99.64	99.82
		Aloe scobinifolia	AJ512307	99.10	99.82
		Aloe inermis	AJ512288	99.10	99.82
		Aloe forbesii	AJ512308	99.10	99.82
		Aloe striata	AJ512310	99.10	99.82
	matK	*Aloe vera*	JQ276402	100.00	100.00
		Aloe nyeriensis	JQ435526	100.00	99.65
		Aloe gneissicola	AY323720	100.00	99.65
		Aloe compressa	AY323721	100.00	99.65
		Aloe harlana	OL689870	98.71	99.52
		Aloe juvenna	AY323717	100.00	99.41

* Only sequences with a GenBank record were considered as reliable and included in the list.

Also the phylogenetic tree indicates that all samples have been *Aloe vera*.

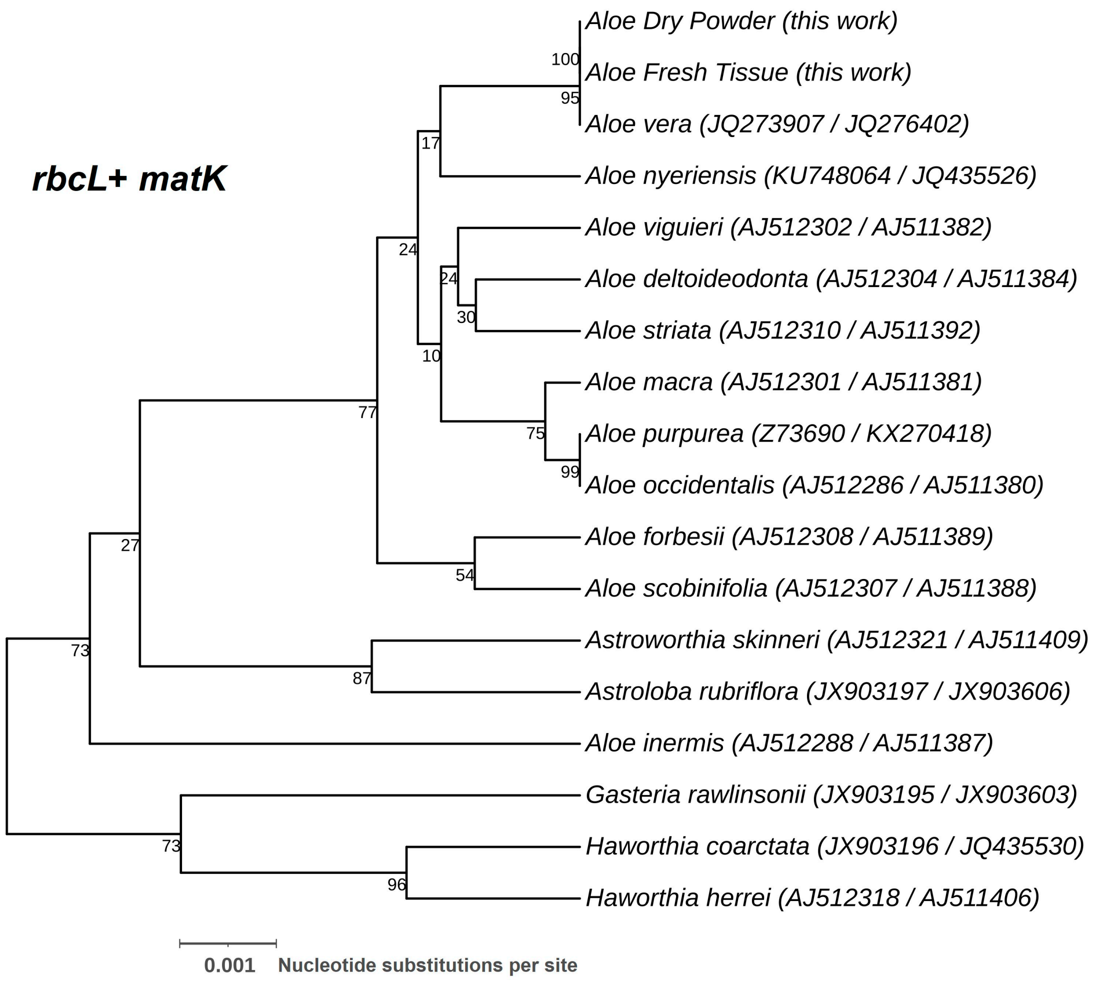

Figure S4: A taxon identification tree for *Aloe vera* and related species created using the UPGMA analysis of Jukes-Cantor nucleotide substitution model based on combined *rbcL* and *matK* sequences. Bootstrap values are shown next to the nodes. Species names are followed by the GenBank ID for *rbcL* and *matK* sequences, respectively. The inferred phylogenetic relationship of the plant samples used in this study is indicated.

References:

1. Kahramanoglu, I.; Chen, C.; Chen, J.; Wan, C. Chemical constituents, antimicrobial activity and food preservative characteristics of *Aloe vera* gel. *Agron.*, **2019**, *9*, id9120831, 18 pp.
2. Femenia, A.; Sánchez, E.S.; Simal, S.; Rosselló, C. Compositional features of polysaccharides from *Aloe vera* (*Aloe barbadensis* Miller) plant tissues. *Carbohydr. Polym.*, **1999**, *39*, 109-117.
3. Minjares-Fuentes, R.; Femenia, A.; Comas-Serra, F.; Rodriguez-González, V.M. Compositional and structural features of the main bioactive polysaccharide present in the *Aloe vera* plant. *J. AOAC Int.*, **2018**, *101*, 1711-1719.
4. Chokboribal, J.; Tachaboonyakiat, W.; Sangvanich, P.; Ruangpornvisuti, V.; Jettanacheawchankit, S.; Thunyakitpisal, P. Deacetylation affects the physical properties and bioactivity of acemannan, an extracted polysaccharide from *Aloe vera*. *Carbohydr. Polym.*, **2015**, *133*, 556-566.
5. Bozzi, A.; Perrin, C.; Austin, S.; Arce-Vera, F. Quality and authenticity of commercial *Aloe vera* gel powders. *Food Chem.*, **2007**, *103*, 22-30.
